# Whole-genome sequence of *Bacillus paralicheniformis* strain R243 isolated from rice (*Oryza sativa* L.) roots

**DOI:** 10.1128/mra.00879-24

**Published:** 2024-10-24

**Authors:** Vladimir K. Chebotar, Maria S. Gancheva, Oksana V. Keleinikova, Maria E. Baganova, Alexander N. Zaplatkin, Veronika N. Pishchik, Elena P. Chizhevskaya

**Affiliations:** 1 All-Russia Research Institute for Agricultural Microbiology, St. Petersburg, Russia; 2 Department of Genetics and Biotechnology, Faculty of Biology, Saint Petersburg State University, Saint Petersburg, Russia; The University of Arizona, Tucson, Arizona, USA

**Keywords:** *Bacillus paralicheniformis*, whole-genome sequence, endophyte

## Abstract

We isolated an endophytic strain R243 and sequenced its genome using the NextSeq 1000 (Illumina) sequencing platform. The sequenced genome contains 4,478 protein-coding genes with a mean GC content of 45.56%. The strain R243 was identified as *Bacillus paralicheniformis*. Gene clusters involved in the synthesis of biocontrol substances were identified.

## ANNOUNCEMENT

*Bacillus* is a large genus of bacteria, mostly found in soil and water. Some species of *Bacillus* promote plant growth and development through the production of phytohormones and siderophores or antibiotics to induce plant resistance against pathogens. Strain R243 was isolated from the endoderm of sterilized roots of rice (*Oryza sativa* L.) variety Boyarin at flowering stage, Proletarskoe experimental farm, near the city Proletarsk (46.718503, 41.659416), in Rostov region, Russia, according to the previous method (1). Strain R243 was grown in LB medium at 28°C for 3 days to get a single colony. The strain was stored in freezer (Sanyo, Japan) at −80°C as cells suspension from a single colony in 20% glycerol. For DNA isolation strain, R243 was grown in LB medium at 30°C overnight. Nucleic acids were extracted using Wizard Genomic DNA Purification Kit (Promega, USA). The resulting DNA was then quantiﬁed using the Qubit 4 ﬂuorometer with the Quant-iT dsDNA BR Assay Kit (Thermo Fisher Scientiﬁc, USA). One hundred nanograms of the extracted DNA was used to prepare a library using the KAPA HyperPlus kit (Roche, Switzerland). After a final purification with KAPA Hyperpure beads (Roche, Switzerland), the library was assessed for size distribution and quality using a high-sensitivity DNA chip (Agilent Technologies, USA) and then quantified using Quant-iT DNA Assay Kit (Thermo Fisher Scientific, USA). The DNA libraries were sequenced using the NextSeq 1000 platform (Illumina, USA), with the NextSeq 1000/2000 P1 Reagents (300 cycles). A 2% PhiX spike-in control was added.

A quality assessment of the reads was conducted using FastQC version 0.11.9 (2). The Illumina sequencing platform produced 1,174,222 paired-end reads. *De novo* assembly was performed using SPAdes version 3.14.1 with the “–careful” option (3). Scaffolds shorter than 500 bp were discarded using a reformat tool from BBMap (https://sourceforge.net/projects/bbmap/). Genome assembly metrics were calculated with QUAST version 5.1.0 (4), samtools coverage tool (5), and BUSCO version 5.2.2 (bacillales_odb10) (6). The obtained genome assembly consists of 15 contigs, spans 4,468,621 bp, with a scaffold N50 of 0.75 Mb and a GC content of 45.56%. The sequencing coverage averaged over contigs was 83. The BUSCO results recovered 99.1% of the Bacillales database. R243 was most closely related to *Bacillus paralicheniformis* reference genome ASM299392v1, with an average nucleotide identity of 99.22% as determined by FastANI (7). The genome was annotated with PROKKA pipeline version 1.14.6 (8), which identified a total of 4,478 protein-coding genes, 61 tRNA genes, and 5 rRNA genes. Biosynthesis clusters were identified using antiSMASH 7.0 (9). The strain R243 was predicted to produce butirosin, fengycin, pulcherriminic acid, schizokinen, bacillibactin, bacitracin, lichenysin, and geobacillin II. Unless otherwise noted, default parameters were used for all software.

## Data Availability

The raw sequences have been deposited in the National Center for Biotechnology Information (NCBI) with SRA number SRR29979682, under BioProject PRJNA1139978. The genome is available under GCF_040965915.1.
